# High correlation of quantitative susceptibility mapping and echo intensity measurements of nigral iron overload in Parkinson’s disease

**DOI:** 10.1007/s00702-024-02856-1

**Published:** 2024-11-01

**Authors:** Adrian Konstantin Luyken, Chris Lappe, Romain Viard, Matthias Löhle, Hanna Rebekka Kleinlein, Grégory Kuchcinski, Sönke Langner, Anne-Marie Wenzel, Michael Walter, Marc-André Weber, Alexander Storch, David Devos, Uwe Walter

**Affiliations:** 1 Department of Neurology, Rostock University Medical Center, Gehlsheimer Str. 20, 18147 Rostock, Germany; 2 Institute of Diagnostic and Interventional Radiology, Pediatric Radiology and Neuroradiology, University Medical Center Rostock, Rostock, Germany; 3 German Center for Neurodegenerative Diseases (DZNE) Rostock/Greifswald, Network of Centers of Excellence in Neurodegeneration (CoEN) Center Rostock, Rostock, Germany; 4 UAR 2014 – US 41 - PLBS - Plateformes Lilloises en Biologie & Santé, University of Lille, Lille, France; 5 INSERM, Centre Hospitalier Universitaire (CHU) de Lille, U1172 - LilNCog - Lille Neuroscience & Cognition, LICEND, University of Lille, Lille, France; 6 Department of Neuroradiology, Centre Hospitalier Universitaire (CHU) de Lille, Lille, France; 7 Institute of Clinical Chemistry and Laboratory Medicine, Rostock University Medical Center, Rostock, Germany; 8 Center for Transdisciplinary Neurosciences Rostock (CTNR), University of Rostock, Rostock, Germany; 9 Neurology and Movement Disorders Department, Reference Center for Parkinson’s Disease, Lille Center of Excellence for Neurodegenerative Disorders (LiCEND), Network of Centers of Excellence in Neurodegeneration (CoEN) Center, Centre Hospitalier Universitaire (CHU) de Lille, Lille, France; 10 Department of Pharmacology, Centre Hospitalier Universitaire (CHU) de Lille, Lille, France

**Keywords:** Magnetic resonance imaging, Parkinson disease, Substantia nigra, Transcranial sonography

## Abstract

Quantitative susceptibility mapping (QSM) and transcranial sonography (TCS) offer proximal evaluations of iron load in the substantia nigra. Our prospective study aimed to investigate the relationship between QSM and TCS measurements of nigral iron content in patients with Parkinson’s disease (PD). In secondary analyses, we wanted to explore the correlation of substantia nigra imaging data with clinical and laboratory findings. Eighteen magnetic resonance imaging and TCS examinations were performed in 15 PD patients at various disease stages. Susceptibility measures of substantia nigra were calculated from referenced QSM maps. Echogenicity of substantia nigra on TCS was measured planimetrically (echogenic area) and by digitized analysis (echo-intensity). Iron-related blood serum parameters were measured. Clinical assessments included the Unified PD Rating Scale and non-motor symptom scales. Substantia nigra susceptibility correlated with echogenic area (Pearson correlation, *r* = 0.53, *p* = 0.001) and echo-intensity (*r* = 0.78, *p* < 0.001). Individual asymmetry indices correlated between susceptibility and echogenic area measurements (*r* = 0.50, *p* = 0.042) and, more clearly, between susceptibility and echo-intensity measurements (*r* = 0.85, *p* < 0.001). Substantia nigra susceptibility (individual mean of bilateral measurements) correlated with serum transferrin saturation (Spearman test, *r* = 0.78, *p* < 0.001) and, by trend, with serum iron (*r* = 0.69, *p* = 0.004). Nigral echogenicity was not clearly related to serum values associated with iron metabolism. Susceptibility and echogenicity measurements were unrelated to PD duration, motor subtype, and severity of motor and non-motor symptoms. The present results support the assumption that iron accumulation is involved in the increase of nigral echogenicity in PD. Nigral echo-intensity probably reflects ferritin-bound iron, e.g. stored in microglia.

## Introduction

Increased iron content in dopaminergic neurons and glial cells in the substantia nigra (SN) has been implicated in the pathophysiology of Parkinson’s disease (PD) (Masaldan et al. 2019; Moreau et al. 2018; Riederer et al. 2021; Ward et al. 2014). Quantitative susceptibility mapping (QSM), an MRI based imaging technique, offers proximal evaluations of iron load in regions of the brain (Wang and Liu 2015). Previous studies using QSM have shown that the tissue magnetic susceptibility correlates well with brain iron, which is elevated specifically in the SN of patients with PD (Acosta-Cabronero et al. 2017; He et al. 2015; Ravanfar et al. 2021). On transcranial sonography (TCS) a characteristic enlargement of SN echosignal (“SN hyperechogenicity”, SN+) is visible at least unilaterally in most PD patients (Becker et al. 1995; Berg et al. 2001, 2008; Walter et al. 2003, 2024). In *post mortem* studies in subjects without extrapyramidal disorders, an increase in SN echogenicity was associated with higher levels of iron, L-ferritin and H-ferritin in the SN (Berg et al. 2002; Gröger and Berg 2012; Zecca et al. 2005). So far, studies comparing SN + on TCS with iron-sensitive MRI data are scarce. A study with PD patients and controls showed contradictory results with no correlation between visual planimetric measurements of echogenic SN area and MRI T2 relaxation times in the whole cohort, but a strong correlation of these measurements for the right SN in the combined group of controls with only SN + and PD patients (Behnke et al. 2009). In another study of PD patients and controls, no correlation was found between the extent of SN + on TCS and susceptibility-weighted MRI measurements, the latter calculated by segmentation-based analysis (Prasuhn et al. 2022). Recently, it was shown in PD patients that enlarged SN areas on TCS and QSM in the caudal midbrain topologically correspond and are located within the SN pars compacta (Ahmadi et al. 2020). Meanwhile, novel software tools allow for digitized quantification of echo-intensity of deep brain structures on TCS (Kozel et al. 2023; Skoloudik et al. 2014; Walter et al. 2014). To further investigate the relationship between TCS and MRI measurements of SN iron content, we here studied the correlation between QSM measures and digitally quantified TCS measures in PD patients. Given that SN iron content influences both, SN echogenicity and susceptibility, we hypothesized that referring TCS and MRI measures are correlated. In secondary analyses, we wanted to explore the correlation of SN imaging data with clinical and laboratory findings. This study is important for biomarker development, as iron accumulation is a marker of cell death by ferroptosis and neuroinflammation (Do Van et al. 2016; Mahoney-Sánchez et al. 2021; Masaldan et al. 2019).

## Methods

### Study design

Between 1st September 2018 and 31st August 2020, PD patients in our movement disorder outpatient clinics were asked to participate in this prospective cohort study. Fifteen patients agreed with participation (four women; age 71.4 ± 9.8 years), five of whom (one woman, four men) were de novo treatment-naïve patients. Three of them underwent imaging examinations (MRI, TCS) of the SN at two time points 9 months apart. Therefore, the data from 18 corresponding MRI and TCS examinations could be analyzed. This study was funded by European funds from Network of Centres of Excellence in Neurodegeneration (CoEN) (PRION-IRON), and was approved by our institutional ethics review board (identifier: A 2018-0085). Written informed consent was obtained from all participants.

### Clinical and laboratory assessments

The severity of PD symptoms was assessed by certified examiners (AKL, HRK) using the Movement Disorders Society-revised version of the Unified Parkinson’s Disease Rating Scale (MDS-UPDRS) (Goetz et al. 2008). Those patients receiving dopaminergic medication were evaluated in the on-medication state. Patients with tremor dominant, postural instability/gait difficulty (PIGD) and appendicular-dominant motor subtypes were identified as described earlier (Stebbins et al. 2013). In addition, non-motor symptoms were assessed on the Beck Depression Inventory (Beck et al. 1961), the Montreal Cognitive Assessment (Nasreddine et al. 2005), the Non-Motor Symptoms Questionnaire (Storch et al. 2010), the Non-Motor Symptoms Scale (Chaudhuri et al. 2007), the REM Sleep Behavior Disorder Screening Questionnaire (Stiasny-Kolster et al. 2007), and the Sniffin’ Sticks 16-odor identification test (Mueller et al. 2006). In all patients, the following serum parameters were analyzed once (at baseline if applicable) at the Institute of Clinical Chemistry and Laboratory Medicine, Rostock University Medical Center: iron, transferrin, transferrin saturation, ferritin, copper, free copper, ceruloplasmin, and zinc. At the time of blood sampling, none of the patients had taken medication known to affect iron or copper metabolism (ferrous sulphate, zinc, iron chelators, *Mucuna pruriens*), nor was there any history or clinical evidence of inflammatory diseases or malignant tumors.

### MRI acquisition and QSM analysis

**Fig. 1:**
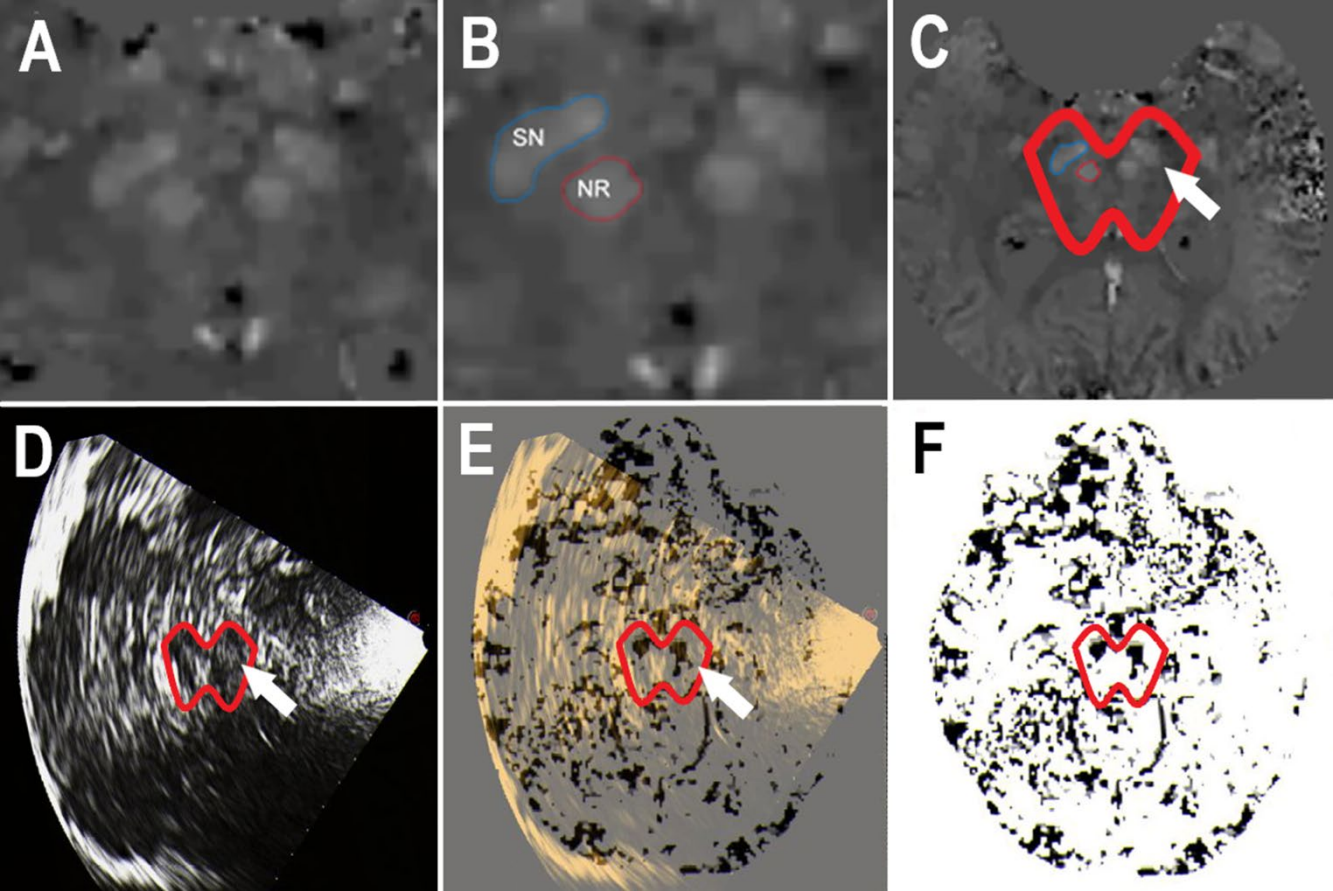
Quantitative susceptibility mapping (QSM) of substantia nigra (SN) and topological correspondence with echo signals. (**A**) MRI susceptibility map of midbrain used for identification of bilateral SN and bilateral red nucleus. (**B**) MRI susceptibility map of midbrain with outlined ROIs of right-sided SN (blue edging) and nucleus ruber (NR; red edging). (**C**) MRI susceptibility map of brain corresponding to the scans shown in (**A**) and (**B**). The midbrain was surrounded (red line) for better recognition (arrow: left-sided SN). (**D**) Transcranial brain sonogram of axial transection through caudal midbrain in a patient with Parkinson’s disease. Enlarged echosignal of bilateral SN is visualized. The midbrain was surrounded (red line) for better recognition (arrow: left-sided SN). (**E**) Exact real-time fusion imaging with transcranial sonography and 3D referenced QSM shows the topological correspondence of SN demarcation by its echogenicity and its susceptibility. The midbrain was surrounded (red line) for better recognition (arrow: left-sided SN). (**F**) Referenced QSM image, with inversion of the image brightness values for better visualization. Note that the QSM plane shown in (**E**) and (**F**) has been reconstructed by the ultrasound system from the imported QSM DICOM 3D dataset according to the ultrasound plane shown in (**D**) and (**E**). The midbrain was surrounded (red line) for better recognition

All participants underwent cranial non-enhanced MRI, using a 3T MRI system (MAGNETOM Skyra Fit, Siemens Healthineers, Erlangen, Germany) and a 32-channel head coil. MRI parameters used for the 3D multigradient echo (mGRE) sequence were as follows: acquisition plane – sagittal; number of slices – 160; slice thickness – 1 mm; field of view – 256 × 256 mm; repetition time – 54 ms; echoes – 6; echo times – 4.22 ms, 9.47 ms, 14.73 ms, 19.98 ms, 25.24 ms, 30.50 ms; flip angle – 15°. QSM was performed following the previously described protocol of the Department of Neuroradiology, CHU Lille, France (Kuchcinski et al. 2023). QSM maps were automatically generated from mGRE data using the morphological-enabled dipole inversion (MEDI) toolbox (de Rochefort et al. 2010; Liu et al. 2011, 2012), which is a MATLAB-based toolbox (version: R2018b; The MathWorks Inc, Natick, Massachusetts, United States). Brain binary masks were created from 3DT1 images (parameters: acquisition plane – sagittal; number of slices – 176; slice thickness – 1 mm; field of view – 256 × 256 mm; repetition time – 2300.0 ms; echo time – 2.98 ms; flip angle – 9°) using volBrain segmentations and co-registered with mGRE images using FSL’s *flirt* function (v6.0.1 fsl.fmrib.ox.ac.uk/fsl). Phase images were unwrapped using an image quality-guided region-growing algorithm. Background field contribution was eliminated using PDF (projection onto dipole fields) and a spherical mean value operation. QSM maps were then calculated from corrected phase images using the MEDI algorithm. The algorithm and the corresponding evaluation parameters of the MEDI-Toolbox were tested using sample data sets provided and adapted according to the specific image characteristics at our imaging site. The parameters lambda, lambda csf, edge and smv were tested in various gradations in order to optimize the image quality. The setting with the lowest number of artefacts and the best resolution for the evaluation of the QSM was then selected. Finally, the following were applied: lambda – 1000, lambda csf – 100, edge – 0.9, smv – 3. The optional functions iFreq and iField correction were activated to achieve better image quality through a higher signal-to-noise ratio (SNR). Further image analysis was performed by a rater unaware to the TCS findings (AKL), using the JiveX software tool (VISUS Technology Transfer GmbH, Germany). For this purpose, the ROIs (here: bilateral SN) were manually marked in the corresponding T1 image data sets (Fig. 1, Panels A-C) and then superimposed on the reconstructed QSM data sets for further analysis. JiveX calculates three-dimensional volumes of interest (VOI) from two-dimensional ROIs and quantifies the mean susceptibility and its standard deviation. The individual susceptibility measures of SN were referenced to the individual average susceptibility of CSF space (Feng et al. 2018; Ravanfar et al. 2021).

### Transcranial sonography and echogenicity analysis

**Fig. 2:**
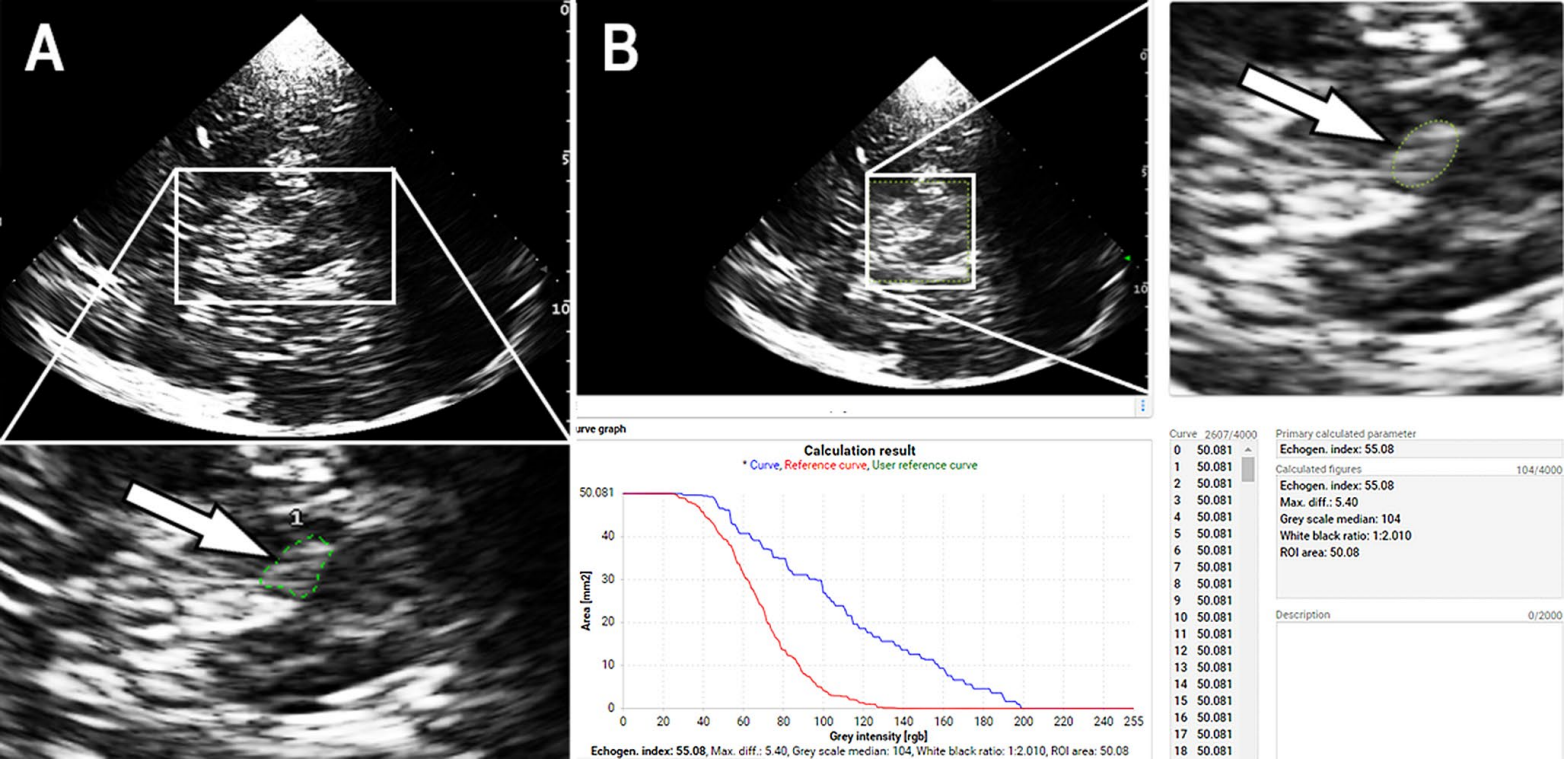
Visualization and measurement of substantia nigra echosignals in Parkinson’s patients. (**A**) Transcranial brain sonogram of axial transection at caudal midbrain level. The lower panel shows the zoomed midbrain in which the substantia nigra echo signals (arrow) on the side ipsilateral to the insonation were manually surrounded for planimetric measurement of echogenic area. (**B**) Screen shot of the MATLAB-based software tool “Cereb B-Mode Assist” used for digitized quantification of substantia nigra echo-intensity. The right panel shows the zoomed midbrain in which an elliptical ROI of 50 mm^2^ was placed to cover the echo signals of the substantia nigra (arrow) on the side ipsilateral to the insonation. The automatic analysis results in the curve of the echo-intensity distribution in the ROI compared to a reference curve determined in healthy controls (lower left panel) and the calculated index value of the total substantia nigra echo-intensity (lower right panel)

TCS was done by an experienced sonographer (UW) in all participants at the same day (± 7 days) as the MRI acquisitions. TCS of SN was performed through the preauricular acoustic bone windows using a high-end ultrasound system (MyLabTwice, Esaote S.p.A., Genova, Italy) equipped with a 2.5-MHz phased-array transducer (PA240) and real-time fusion imaging technology (Virtual Navigator). The ultrasound system settings were as follows: view – 3, size of aperture – 89°, dynamic range – 6, dynamic compression – 2, persist – 7, enhance – 3, density – 2, focuses – 1, gray map – 5, mechanical index – 1.0. SN ipsilateral to insonation was assessed from both sides. The topological correspondence of SN-ROIs in the referenced QSM maps (see previous paragraph for details) and TCS images of the caudal midbrain, demonstrated in a previous study (Ahmadi et al. 2020), was verified here by applying a real-time fusion imaging technique in a randomly selected study participant (Fig. 1, Panels D-F). For this purpose, the DICOM volume dataset of the referenced high-contrast QSM maps was loaded into the ultrasound system and co-registered with the subject’s head position; the image registration and fine-tuning procedures were carried out to exactly superimpose the MRI and TCS images as described earlier (Walter et al. 2016). Planimetric measurements of SN echogenic size were performed in all study participants on axial scans automatically after manually circling the outer circumference of SN’s echogenic area (Fig. 2, Panel A). With the ultrasound system applied, SN echogenic sizes of < 0.24 cm² are classified as normal, sizes of ≥ 0.24 cm² representing upper 25% percentile in normal population as hyperechogenic (SN+), and echogenic sizes of ≥ 0.30 cm² representing upper 10% percentile in normal population as marked SN+ (Walter and Skoloudik 2014). To quantify the echo-intensity of SN, off-line digitized image analysis was performed using the validated MATLAB-based software tool “Cereb B-Mode Assist” (MEDDIAG Cereb B-Mode Assist, Olomouc, Czech Republic) (Skoloudik et al. 2014; Kozel et al. 2023). This tool pre-assesses the overall image quality before starting the analysis of distinct structures, yielding a normalized echo-intensity measure of the target structure in the referring ROI (Fig. 2, Panel B).

### Statistical analyses

To test our main hypothesis, QSM susceptibility measures and TCS measures (planimetric measurements of SN echogenic size, digitized measurement of SN echo-intensity) were compared using the Pearson correlation test. For the secondary exploratory studies, the Spearman test was employed to compare the imaging data (QSM, TCS; individual mean values of the bilateral SN measurements) with clinical and laboratory data. As eight clinical (age, duration of motor symptoms, scores on the motor part of the MDS-UPDRS, BDI, MoCA, NMSS, RBDSQ and SS-16) and eight laboratory variables were tested for the secondary correlation analyses, we applied a Bonferroni correction for multiple comparisons, where *p* ≤ 0.003 indicated statistical significance. For the analyses the Statistical Package for the Social Sciences (IBM SPSS Statistics for Windows, IBM Corp., Version 27.0, Armonk, NY) was used.

## Results

### Clinical and laboratory findings

The clinical and laboratory data are summarized in Tables 1 and 2. Nine patients were classified as PIGD, four as tremor-dominant, and two as appendicular-dominant motor subtype of PD.

**Table 1:** Demographic and clinical findings in the study participants (4 women, 11 men)

Feature^1^	Mean ± SD	Minimum	Maximum
*Demographic*			
Age, y	71.4 ± 9.8	56	85
Motor symptom duration, y	7.1 ± 6.4	0.8	19.7
Dopaminergic treatment duration, y ^2^	8.6 ± 5.1	1.4	19.5
Levodopa equivalent daily dose, mg	459 ± 440	0	1325
*Motor symptom severity*			
Hoehn and Yahr stage	2.5 ± 0.7	2	4
MDS-UPDRS, part 1, score	11.6 ± 7.3	1	27
MDS-UPDRS, part 2, score	16.1 ± 6.2	5	28
MDS-UPDRS, part 3, score	33.3 ± 13.1	19	71
MDS-UPDRS, part 4, score	3.0 ± 4.9	0	16
*Non-motor symptom severity*			
BDI, score	11.1 ± 9.3	0	35
MoCA, score	23.8 ± 2.7	19	29
NMSS, score	53.9 ± 43.8	0	164
RBDSQ, score	6.7 ± 3.8	1	13
SS-16, score	5.6 ± 3.6	0	12

BDI denotes Beck Depression Inventory; MDS-UPDRS, Movement Disorders Society-Unified Parkinson’s Disease Rating Scale; MoCA, Montreal Cognitive Assessment; NMSS, Non-Motor Symptoms Scale; RBDSQ, REM Sleep Behavior Disorder Screening Questionnaire; SS-16, Sniffin’ Sticks 16-odor identification test

^1^ Demographic and clinical data were collected in the 15 patients once at baseline

^2^ Treatment duration in the 10 patients receiving dopaminergic medication

**Table 2:** Laboratory findings in the study participants (4 women, 11 men)

Parameter ^1^	Mean (SD)	Minimum	Maximum
Iron, µmol/L	13.1 (5.5)	3.8	21.8
Transferrin, g/L	2.5 (0.5)	1.7	3.6
Transferrin saturation, %	22.5 (10.9)	8.9	44.2
Ferritin, µg/L	194 (132)	14	465
Copper, µg/L	1003 (251)	600	1648
Free copper, µg/L	36.1 (57.1)	6.4	210
Ceruloplasmin, g/L	0.31 (0.05)	0.24	0.42
Zinc, µmol/L	9.55 (3.74)	4.96	19.40

^1^ Laboratory parameters were estimated in the 15 patients once at baseline

### TCS findings

SN echogenic areas could be visualized and measured bilaterally in all patients. Marked SN + was found at least unilaterally in 10 (67%) patients, moderate SN + in another four (27%), and bilaterally normal SN echogenic area in one. Planimetric SN echogenic area measurements correlated with digitized echo-intensity measures (Pearson correlation, *r* = 0.72, *p* < 0.001).

### Correlation between QSM and TCS measures

**Fig. 3:**
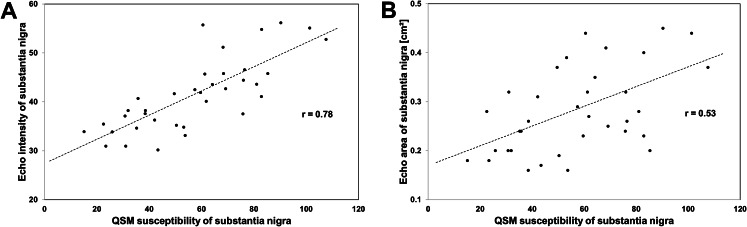
Diagrams showing the relationship between susceptibility and echogenicity measurements of the substantia nigra in Parkinson’s patients. (**A**) Relationship between susceptibility and digitally analyzed echo-intensity of the substantia nigra (Pearson test, *p* < 0.001). (**B**) Relationship between the susceptibility and the planimetrically measured echogenic area of the substantia nigra (*p* = 0.001)

QSM susceptibility correlated with digitally assessed SN echo-intensity (Pearson correlation, *r* = 0.78, *p* < 0.001; Fig. 3, **Plot A**) and planimetric SN echogenic area (*r* = 0.53, *p* = 0.001; Fig. 3, **Plot B**). Individual asymmetry indices, calculated for each modality by dividing the individual larger value by the smaller of the bilateral SN measurements, were found to correlate between susceptibility and echogenic area measurements (*r* = 0.50, *p* = 0.042) and, more clearly, between susceptibility and echo-intensity measurements (*r* = 0.85, *p* < 0.001).

### Correlation between QSM and laboratory measures

**Fig. 4:**
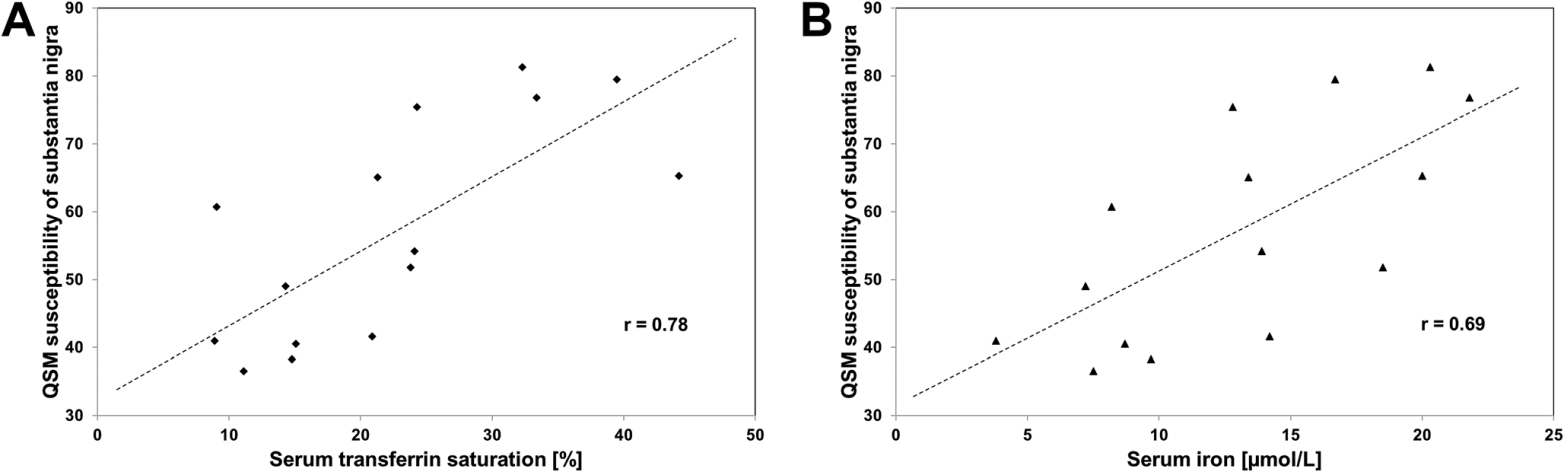
Diagrams showing the relationship between the measurement of circulating iron levels and the susceptibility of the substantia nigra in Parkinson’s patients. (**A**) Relationship between serum transferrin saturation and substantia nigra susceptibility (individual mean of bilateral measures; Spearman test, *p* < 0.001). (**B**) Relationship between serum iron and substantia nigra susceptibility (individual mean of bilateral measures; *p* = 0.004, not significant after Bonferroni correction with significance level set at *p* ≤ 0.003)

QSM susceptibility (individual mean of bilateral measurements) correlated with serum transferrin saturation (Spearman test, *r* = 0.78, *p* < 0.001; Fig. 4, **Plot A**) and, by trend, with serum iron (*r* = 0.69, *p* = 0.004; Fig. 4, **Plot B**), however with none of the other serum parameters (each, *p* > 0.1).

### Correlation between TCS and laboratory measures

SN echo-intensity (individual mean of bilateral measurements) correlated, by trend, with serum transferrin saturation (Spearman test, *r* = 0.62, *p* = 0.013). No correlation was found between planimetric SN echogenic size (individual mean of bilateral measurements) and serum parameters (each, *p* > 0.1), nor between SN echo-intensity (individual mean of bilateral measurements) and the remaining serum parameters (each, *p* > 0.07).

### Correlation between imaging and clinical data

Neither SN echogenic area nor SN echo-intensity measures nor QSM data were found to correlate with age, disease duration, nor with any of the symptom severity scores (each, *p* > 0.05).

## Discussion

Our present findings show a clear correlation between the tissue magnetic susceptibility and the digitally measured echo-intensity of the SN in patients with PD. There is also a correlation between the tissue magnetic susceptibility and visual planimetric measurements of the SN echogenic area, but it is less strong. We found no correlation of SN susceptibility or SN echogenicity with clinical findings such as PD duration or symptom severity.

This is, to our best knowledge, the first study directly comparing QSM susceptibility and SN echogenicity measures. Strengths of the present study are its prospective design and independent assessment of susceptibility and echogenicity by blinded raters. Potential limitations comprise the influence of temporal bone thickness on TCS image quality and the use of a locally adapted protocol for susceptibility analysis. We aimed at minimizing these influences by maintaining strict quality standards for MR and TCS image acquisition and by using uniform measurement protocols for all study participants.

Non-heme iron in the brain is mainly bound to ferritin, which makes up the greatest contribution to the tissue magnetic susceptibility among all iron compounds. Neuromelanin is another iron-containing complex that is abundant in the SN pars compacta (SNc) and constitutes a main source of magnetic susceptibility in the SN (Ravanfar et al. 2021). In PD there is a loss of pigmented cells, and an overall reduction in the amount of melanin within remaining cells (15%) in SN because of a more severe (80%) loss of heavier pigmented cells, leading to massive disturbance of the iron–neuromelanin interaction and iron release from neuromelanin (Fasano et al. 2006; Riederer et al. 2021). This excess iron is increasingly bound by ferritin which itself causes enhanced susceptibility on MRI. On the other hand, animal experiments and post-mortem analyses of brain tissue of non-symptomatic subjects have shown that SN hyperechogenicity (SN+) is associated with increased local iron and ferritin levels, but a reduced neuromelanin content (Berg et al. 2006; Zecca et al. 2005). The high correlation of SN echo-intensity on TCS and QSM susceptibility on MRI found in the present study suggests that both measures mainly reflect ferritin-bound iron in the SN. This fits well to the findings of a recent study combining quantitative 3D iron histology and biophysical modeling with quantitative MRI on *post mortem* human brain tissue, suggesting higher relative sensitivity of R_2_* sequences to neuromelanin-bound iron, and of QSM bulk susceptibility to ferritin-bound iron (Brammerloh et al. 2021). Refined assessment of different iron-sensitive MRI parameters (R_2_*, R_2_, QSM susceptibility) might therefore enable the discrimination of neuromelanin- and ferritin-bound iron pools in the SN, and allow for a more reliable PD staging. Recently, the simultaneous MR acquisition of neuromelanin-sensitive and iron-sensitive (QSM) SN imaging data has been proposed for monitoring PD progression (Gaurav et al. 2021; He et al. 2021; Martínez et al. 2023). These new approaches could resolve the conflicting results of previous studies on SN magnetic susceptibility, some of which suggest a correlation with the duration of PD (He et al. 2015; Du et al. 2016), while others do not (Ghassaban et al. 2019; Lancione et al. 2022). The presently found high correlation of QSM susceptibility, which appears to be rather stable during the course of PD (Ghassaban et al. 2019; Lancione et al. 2022), and echogenicity on TCS, is in accordance with the earlier finding of long-term stability SN echogenicity and independence from disease severity in PD patients (Berg et al. 2005; Behnke et al. 2013; Walter et al. 2007). Of note, asymmetry of individual SN susceptibility correlated well with asymmetry of SN echo-intensity. It has been shown earlier in TCS and MRI-QSM studies that imaging asymmetry of nigral iron deposition is related to motor laterality in PD (Azuma et al. 2016; Berg et al. 2001; Guan et al. 2020; Toomsoo et al. 2019; Walter et al. 2006). However, the neuropathological correlates of QSM changes in the SN of PD patients are not yet fully understood. QSM changes may also reflect chronic neuro-inflammation (e.g. with iron-loaded microglia) or myelin loss, and not necessarily just iron accumulation (Rua et al. 2024). In addition, neuro-inflammatory processes in the SN reflected by an increase in free water on MRI may differ from those reflected by increased susceptibility (Chen et al. 2023).

In this study, a strong correlation was found between measures of circulating iron and QSM susceptibility of SN in PD patients. Earlier studies did not investigate this systematically, or yielded insignificant results (Xu et al. 2023). Our finding of a positive correlation between serum iron and nigral susceptibility may be surprising in view of the previously proposed negative correlation between serum iron level and the risk of PD (Chen et al. 2024; Pichler et al. 2013), and the report of lower serum iron levels in patients with PD compared with age- and sex-matched controls (Jiménez-Jiménez et al. 2021). However, the findings of another meta-analysis suggest higher serum iron levels in PD patients as compared to healthy controls (Jiao et al. 2016). While in the present study no clear relationship between SN echogenicity and measures of circulating iron was found, in an earlier study we observed rather a negative correlation between SN echogenicity and serum iron (Walter et al. 2012). Hereditary disturbances of iron metabolism, e.g. neurodegeneration with brain iron accumulation, may cause increase of SN echogenicity (SN+) (Habibi et al. 2024; Kostić et al. 2012; Liman et al. 2012). The possible link between serum and CSF parameters related to iron metabolism and the degree of SN + in Parkinson’s patients, as implied by the results of previous studies (Li et al. 2020; Walter et al. 2012; Ying et al. 2023; Yu et al. 2018), needs further investigation.

It should be emphasized that SN + is probably not only influenced by iron levels, but also by other processes such as neurotoxin-induced damage and microglia activation (Subramanian et al. 2010; Berg et al. 2010). Our finding that both, SN susceptibility and serum transferrin saturation, correlate with SN echo-intensity rather than SN echogenic area suggests that SN echogenic area may be influenced to a greater extent by processes other than iron content. It also remains to be clarified whether or not SN + in Parkinson’s patients can change to some extent over time (Tomsoo et al. 2019; Weise et al. 2009). The pending analysis of TCS data, collected at baseline and after six months of treatment with deferiprone versus placebo in a sub-cohort of Parkinson’s patients in the FAIRPARK II study (Devos et al. 2022), may provide further insights into the changeability of SN + in PD.

## Conclusions

The present finding that QSM susceptibility correlates with SN echo-intensity in Parkinson’s patients supports the hypothesis that iron accumulation plays a crucial role in the development of SN+. SN echo-intensity probably reflects ferritin-bound iron, e.g. stored in microglia. In comparison to SN echo-intensity, the echogenic area of the SN could additionally be influenced by other pathological changes in PD. TCS of SN can easily be included in the clinical diagnosis and follow-up routine for patients with PD. Further studies are warranted to investigate the potential for therapy-induced reduction of SN + in PD.

## Data Availability

All data are available from the corresponding author on reasonable request. The request necessitates that the purpose of data re-analysis is in line with the study aims as approved by the ethics review board (see text) and participants consent.
